# A single-session Mindfulness-Based Swinging Technique vs. cognitive disputation intervention among women with breast cancer: A pilot randomised controlled study examining the efficacy at 8-week follow-up

**DOI:** 10.3389/fpsyg.2022.1007065

**Published:** 2022-10-20

**Authors:** Ozan Bahcivan, Jose Gutierrez-Maldonado, Tania Estapé

**Affiliations:** ^1^Department of Clinical Psychology and Psychobiology, Faculty of Psychology, University of Barcelona, Barcelona, Spain; ^2^Psiko-Onkologlar Dernegi (Turkish Psycho-Oncological Association), Izmir, Turkey; ^3^FEFOC Foundation, Barcelona, Spain

**Keywords:** mindfulness, MBST^®^, Mindfulness-Based Swinging Technique, cognitive disputation, breast cancer, anxiety, self-efficacy, adherence-to-treatment

## Abstract

**Objective:**

Previously Mindfulness-Based Swinging Technique (MBST)'s immediate efficacy for overcoming psychological concerns has recently received empirical support, yet its longer-term efficacy needed to be evaluated among women with breast cancer. The objective of this study was to assess and report the efficacy of MBST intervention among breast cancer patients for hopelessness, anxiety, depression, self-efficacy, oxygen (SpO_2_) intensity, and heart rate-beats per minute (HR-bpm) at an 8-week period.

**Method:**

The State-Trait Anxiety Inventory, The Emotion Thermometer, Hospital Anxiety and Depression Scale, Self-Efficacy for Managing Chronic Disease, and Beck's Hopelessness Scale were used for assessing the intervention's outcome; 149 BC patients were randomly assigned into two groups (equal-mean-age, *p* = 0.262). The participants in the control group (CG, *n* = 73) received Cognitive Behavioural Therapy (CBT)-Cognitive-Disputation (CBT-CD) for 20 min, and intervention group (IG, *n* = 76) received MBST intervention. No additional psychological interventions were given between week-1 and week-8.

**Result:**

Outcomes of the 8-week post-treatment follow-up exhibited significantly higher improvements in all evaluated-measurements for CG, and some for IG with large effect size in the following: anxiety (CG *p* < 0.05, r = 0.57; IG *p* < 0.05, r = 0.44) and depression levels (CG *p* < 0.05, r = 0.43). It increased self-efficacy for managing disease (CG *p* < 0.05, r = 0.49; IG *p* < 0.05, r = 0.41) and hopefulness (CG *p* < 0.05, r = 0.59; IG *p* < 0.05, r = 0.46), and saturation levels measured by pulse-meter/oximeter (CG *p* < 0.05, r = 0.49; IG *p* < 0.05, r = 0.32).

**Conclusions:**

Both CBT-CD and MBST have been found to be efficacious interventions to shorten the psychotherapy duration for reducing clinical anxiety and hopelessness as well as increase self-efficacy for BC women. This may have a distinct clinical importance for supporting BC patient's adherence-to-treatment since CBT-CD could be an alternative technique to MBST as a brief intervention. In future studies, the effectiveness of MBST through adapting to virtual reality and other online delivery methods should be examined.

## Introduction

Cancer is accepted as one of the main global public health concerns and is listed as the second prominent cause of death (Siegel et al., 2021). In 2020, 2.3 million women were diagnosed with breast cancer with a total of 685,000 deaths worldwide (Sung et al., 2021). In fact, breast cancer is reported as the most prevalent cancer type among women, and the statistics are similar in Turkey (Kilickap et al., 2017; World Health Organisation, 2020). The coronavirus disease 2019 (COVID-19) adversely affected psycho-oncological and medical treatment of breast cancer patients (Cheli et al., 2022). This is mainly because of the reduced access or temporary closure of the health care facilities to avoid the spread COVID-19 (Yabroff et al., 2022) or patients delayed their psychological and physical treatment or/and check-ups due to their personality traits (Cheli et al., 2022). According to Ping et al. (2020), mental health professionals at research institutions primarily sought and developed brief psychological interventions to reduce patient's contact and hospital visit times during the pandemic. Studies in the current field demonstrated that 40–50% of patients with breast cancer were identified to have mental health disorders, which involved mood disorder, anxiety disorder, adjustment disorder, and mental disorders that were caused by medical conditions (Youlden et al., 2014; Sun et al., 2019). Therefore, providing effective psycho-oncological techniques to alleviate anxiety, stress, and hopelessness is highly desirable among breast cancer patients (Wolanin, 2021).

Kapogiannis et al. (2018) and Palesh et al. (2018) stated that psychosocial interventions can be utilised in order to ease the side effects of medical treatment of cancer. Yet, the mental and behavioural health clinicians came across various difficulties which needed extensive alterations in the area of health care, such as the duration of the psychotherapy session (Sperry and Binensztok, 2019). They stated that many individuals who need medical care also had psychological conditions that were induced, worsened, or prolonged by their medical status which required a better treatment model. Therefore, longer and lengthy therapy sessions may not be practical for breast cancer patients who receive oncological treatments (Teo et al., 2019).

On the other hand, heart rate (HR) is largely under the control of peripheral nerve system activity during relaxation, activated by deep breathing (Palma et al., 2020). A study conducted by Mallorqui-Bague et al. (2016) argued that there is an association between interpreting an event as stressful that changes in cardiovascular activity, for example, increasing HR beats per minute (bpm) and the density of anxiety. Moreover, the results show that anxiety sensitivity is more common among females than males (Norr et al., 2015; Trotman et al., 2019). Therefore, HR may be considered as a suitable psychophysiological indicator to measure anxiety and stress (Lorca et al., 2019). There is a continuously growing body of literature on mindfulness that demands up-to-date reviews regularly for breast cancer population (Cifu et al., 2018).

### Brief mindfulness-based interventions

There has been a great increase in the development of mindfulness-based interventions (MBI), which are still being developed even today, starting from the beginning of the 2000's (Van Dam et al., 2018). Mindfulness is a term covering a wide range of subjects that identify many practises, processes, and attributes. Mindfulness is mainly described concerning the content of attention, awareness, retention, and acceptance (Van Dam et al., 2018). Yet, Analayo (2019) stated that the definitions of mindfulness are subject to debate and are diverse. Contemplative traditions and scientific disciplines of psychology, medicine, and education are combined in mindfulness-based practises (Baer, 2019; Birtwell et al., 2019). There has been a significant change in the period of MBIs in order to correspond with short training programs, which could consist of only four 20-min-long sessions (Zeidan et al., 2015). Shorter psychological treatments mean a reduction in health care expenses. Present psychotherapy protocols are prone to guide treatments concerning the best price in the treatment of anxiety disorders (Otto et al., 2012). Sanada et al. (2017) stated that utilising different mindfulness techniques, which involve short interventions and/or a 15-min recording of mindfulness-based exercise positively affects cancer patients significantly (Tang et al., 2015). These form crucial improvements in many health indicators (Lorca et al., 2019). Results indicated that MBIs provide multiple health benefits over a short period of time in clinical participants (Solhaug et al., 2019). These benefits include enhanced well-being and decreased depression, anxiety, stress, and burnout (Burton et al., 2017). There is some evidence that mindfulness interventions and medical disease management complete each other in treating individuals with physical illness by comforting psychological distress and improving wellbeing (Janusek et al., 2019; Russo, 2019; Zimmermann et al., 2020). Yet, individuals who were on the course of active cancer treatment had difficulty with mindfulness practise. This is due to the possible side effects of the treatment which resulted in fatigue, which made mindfulness practise more difficult (Toivonen et al., 2020).

According to a systematic review in mindfulness (Xunlin et al., 2020) as well as another systematic review in Tai Chi/qigong (Wayne et al., 2018) among cancer patients, it was found that there were remarkable enhancements in their anxiety and depression symptoms. The perceived stress and anxiety experienced among cancer patients are observed through saturation level (SpO2), which is considered as a psychophysiological indicator (Ng et al., 2016; Beng et al., 2019). In fact, a study conducted by Xue et al. (2020) indicated that after breathing-based mindfulness practises, the saturation level (SpO2) increased among participants. Yet, it is noted by Carlson et al. (2014), taking individuals' choices into consideration plays a role in autonomous decision-making, allowing for higher perceived control when it comes to health results. This brings about the best efficiency of the intervention (Oberoi et al., 2021). According to Social Constructivist Theory (SCT), one gains knowledge by means of social and individual group interactions (Conrad and Barker, 2010). From the social constructionist point of view in regard to illness, increasing the patient's participation and decreasing apprehension, the content of their illness as well as diagnosis by including patient's personal and cultural background is important. Thus, patients' participation, apprehension, and effort in handling their diagnosis may be positively affected (Oberoi et al., 2021).

### Brief cognitive behavioural interventions

Cognitive Disputation (CD) can be defined as a cognitive behavioural intervention, which aims to achieve aiding individuals in recognising their irrational thinking pattern by using logic (Sperry and Binensztok, 2019). The whole aim of the provider is to train the patient by disputing their illogical beliefs by adopting a logical approach so that the patient can confront their thoughts on their own. One way of doing this is through Socratic questioning, which is on the basis of Aaron Beck's cognitive therapy, which involves detecting logical errors or cognitive distortions; that can be helpful for disputing thoughts in a shorter duration (Overholser, 2011). Often, they could be challenging as they are acceptable and automatic, precise and distinct, as well as unusual and useless (Beck, 1979). Despite the fact that these thoughts can make one's life quite difficult, patients see these thoughts as logical and reasonable, although they lead to emotional and behavioural disruptions (Lam and Cheng, 1998; Akkoyunlu and Turkcapar, 2013; Sperry and Binensztok, 2019).

Thus, the purpose of this randomised pilot study was to follow-up on those results reported in Bahcivan et al. (2022), and further report on MBST's efficacy at 8 weeks. Therefore, it is hypothesised that; the 20-min long MBST intervention will aid patients' perceived self-efficacy by improving their hope in regard to their cancer treatment and lessen anxiety as well as boost their oxygen (SpO_2_) intensity and slow down their heart rate (bpm).

## Materials and methods

### Design

This is a pilot randomised controlled study trial registered in the United States (U.S.) National Library of Medicine Registry, ClinicalTrials.gov *identifier* NCT03985267. This pilot randomised controlled trial was performed by carefully following the *CONSORT* (Consolidated Standards of Reporting Trials) *2010 guidelines statement extension to randomised pilot and feasibility trials* (Eldridge et al., 2016). All patients included in this pilot study have agreed and signed informed consent.

### Participants

Inclusion criteria were applied to women participants with breast cancer in this study, which was previously published by Bahcivan et al. (2022): (a) women diagnosed with breast cancer, (b) who can consent, (c) native Turkish speakers, (d) currently under cancer treatment, (e) score at least 16 points for Hospital and Depression scale (8 for anxiety, 8 for depression), (f) score maximum 7 points for Self-Efficacy for Managing Chronic Disease (in overall), (g) score at least 4 points for Beck's Hopelessness Scale (in overall), and (h) score at least 40 points for State-Trait Anxiety Inventory.

**Figure 1:**
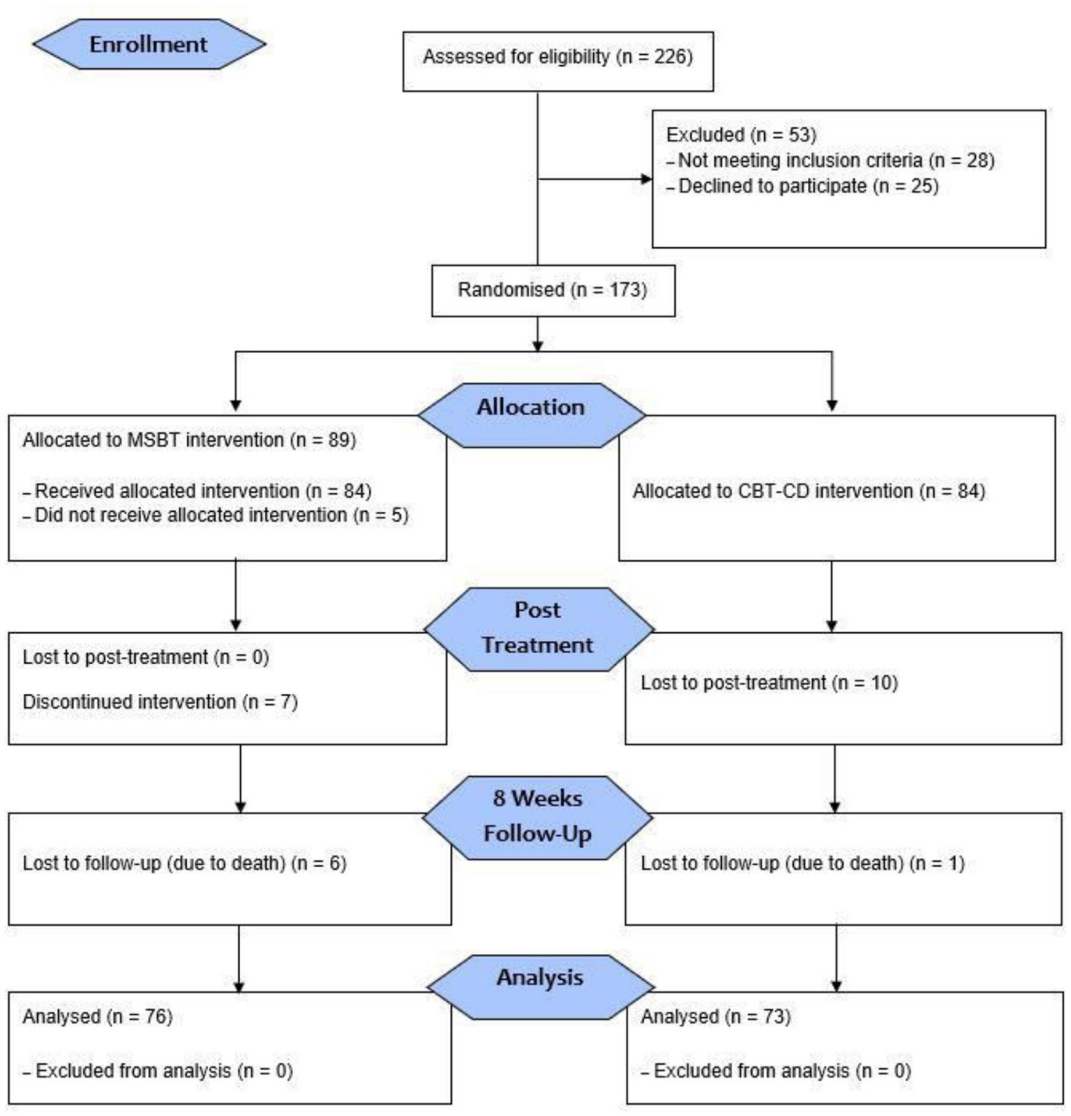
Flow diagram displaying the participants' selection, distribution, post-treatment, and follow-up.

Among those 173 eligible participants in week-1, 84 were allocated to control group, and 89 were assigned to the experimental group. The follow-up period was 8 weeks from the post-treatment period. After the 8-week follow-up, a total of 149 participants were included and analysed (Figure 1). A total of 82 participants completed the MBST intervention in week-1, 76 participants completed the 8-week follow-up. Since 85.4% of the participants completed the MBST intervention, the dropout rate was very low (14.6%). The participants were registered from March 2019 to August 2021.

### Intervention

The intervention called “Mindfulness Based Swinging Technique (MBST)” was applied by the instructor to the eligible participants right after the psycho-social assessments which were approximately 20-min long. The MBST intervention period included a specific guided imagery for swinging practise and a breathing exercise as formerly explained (Bahcivan et al., 2018, 2022). Additionally, a brief psychoeducation about the nature of mindfulness and directives for the intervention was given for approximately 5-min by the instructor before the MBST intervention commenced. Participants carried out the same psycho-social assessment in the control group but did not take part in the MBST, instead they undertook 20 min of CBT-CD, which was previously described (Horne and Watson, 2011; Sperry and Binensztok, 2019; Bahcivan et al., 2022). The protocol of CBT-CD treatment is further described by Sperry and Sperry (2017). Since participants' allocation and taking part in the interventions happened on the same day, there was no time interval. After 8 weeks, the same participants from both groups were required to complete the equal follow-up psycho-social assessments, which were given in their initial participation but did not receive any of the above-mentioned interventions at this time.

### Outcome assessment

The psycho-social measuring tools for anxiety, distress, self-efficacy, hopelessness, and depression symptoms were completed by the participants, as well as receiving measurements of heart rate (bpm) and oxygen saturation (SpO_2_) level just before commencing the intervention, and just after completing the intervention. After 8 weeks from the initial participation, patients were invited once again to receive the same psycho-social assessments including measurement of heart rate (bpm) and SpO_2_ level. All of the measuring tools were self-administered at the hospital and psychological consultancy centre on all occasions.

### Outcomes

#### Distress and anxiety symptoms

The distress and anxiety symptoms were assessed by using the Emotion Thermometer (ET) and State-Trait Anxiety Inventory (STAI). The ET developed by Mitchell et al. (2010), which has 5-visual individual analogue scales that measure anxiety, distress, depression, and anger, and the final outcome domain called “need for help” was applied among cancer population; 0 (none) to 10 (extreme) ratings for each of the four emotional area scales were used. An optimal balance between specificity and sensitivity was found by Mitchell et al. (2010). Participants were asked to pick the best indicating number for their level of emotion.

Bahcivan and Eyrenci (2018) adapted the Turkish version of ET. The overall Cronbach's alpha of 0.87 was reported in their adaptation study. For depression thermometer, the optimal cut-off score was 4, and for anxiety and distress thermometers of ET, it was 5 for both which yielded the optimal sensitivity and specificity values (sensitivity scores: 0.86, 0.75, and 0.73 and specificity scores: 0.70, 0.68, and 0.67, respectively). The scale was found to be an acceptable and practical tool for psychological distress screening among cancer patients by Bahcivan and Eyrenci (2018).

The state anxiety and trait anxiety were measured by STAI, which consisted of two 20-item subscales measures (Spielberger et al., 1983). The STAI is self-administered on a four-point scale for each item, patients were required to indicate how they felt for each of the 40 items. The scores for each of the subscales ranged from a minimum of 20 to a maximum of 80, the higher scores suggest the greater psychological anxiety. An internal consistency was found to be 0.95. Oner and Le Compte (1983) adapted The STAI in Turkish language and culture. They found that STAI is a valid and reliable psychometric tool and appropriate for the cancer population. The internal reliability was indicated as 0.72, and test–retest reliability was reported as 0.86.

#### Depression symptoms

The depression and anxiety symptoms were assessed by Hospital Anxiety and Depression Scale (HADS). Zigmond and Snaith (1983) developed the HADS that consisted of 14 questions. Aydemir et al. (1997) completed the Turkish adaptation studies for their validity and reliability. The scores ranged from 0 (strongly disagree) to 3 (strongly agree) on a Likert scale. The cut-off points of 8 for both anxiety and depression scores were used, respectively, to adapt to cultural norms (Miljanović et al., 2017). The HADS tool is commonly used in oncology settings for its validity and reliability reasons (Clover et al., 2020).

#### Self-efficacy

In order to assess self-efficacy, the Self-Efficacy for Managing Chronic Disease (SEMCD) scale was used. It is specifically designed to test for the management of chronic diseases. Lorig et al. (2001) developed and validated the 6-item version of the SEMCD. It consisted of a 10-point Likert-type scale with “1” being the “not at all confident” and with “10” being the “totally confident.” The higher score suggests an increase in management in self-efficacy about their chronic disease. Incirkuş and Nahcivan (2020) adapted the 6-item version to the Turkish language and culture. The Cronbach alpha value for the reliability was reported as 0.95 for the SEMCD-total score and was found to be a reliable and valid tool for the clinical practise among Turkish cancer patients (Ozkaraman et al., 2019).

#### Hopefulness

To assess hopefulness, the Beck's Hopelessness Scale (BHS) was used. Beck et al. (1974) developed the initial scale, they found the internal consistency to be high with Cronbach's alpha being 0.85. Durak and Palabiyikoglu (1994) adapted BHS into the Turkish language. The Cronbach alpha internal consistency coefficient of scale was 0.85, two-half reliability coefficient was 0.85 and test–retest reliability was 0.74. According to Kavak Budak et al. (2021), BHS could be used among Turkish cancer patients.

#### Sample size

During the study planning, the sample size was calculated (see supporting information). A minimum of 45 participants for each group was determined for the necessary subjects' numbers to be able to reject the null hypothesis. The population means of the experimental and control groups are equal with probability (power) 0.9. The type-I error probability associated with this test of the null hypothesis is 0.05.

#### Randomisation, allocation, and concealment

Initially, the eligible participants signed the informed consent form and randomly registered either the experimental (MBST intervention) or the control group (CBT-CD intervention) (Figure 1). The registration and random allocation sequence, as well as the qualified 85 participants, were from the EgeMed Hospital in Aydin, Turkey, and 88 participants were recruited from Ozel Oz Psikoloji Aile Danisma Merkezi (Oz Psychology Family Counselling Centre) in Izmir, Turkey, then randomisation was generated by an authorised health care personnel from the recruiting centre. The numbers signify the patients' admittance sequence. Randomisation was completed through a computer-generated list of random numbers. The study results were evaluated by self-administered questionnaires with the assistance of a researcher psychologist. The participants who lost to post-treatment were discontinued from the allocated intervention; this is due to their pre-existing medical discomfort. The 8-week follow-up procedure was conducted by re-inviting the participants *via* phone call which was provided in the initial intake by the participants. A total of 7 participants lost to follow-up due to death, reported by a next of kin of the patient.

### Statistical analysis

This study is a continuation of the research conducted by Bahcivan et al. (2022). Although initially this study was carried out with a total of 156 patients (74 in control group, and 82 in intervention group) including pre-post comparisons results in the aforementioned research; while in this continuation study, it was carried out with a total of 149 patients (73 in control group, and 76 in intervention group) only who completed the follow up at 8 weeks. Therefore, the comparisons of pre-post and post-follow-up results are based on the completion of the 8-week point by the patients. The patient's characteristics in this current study were described with frequency and percentages. The Chi-square test was examined whether there was a difference among these categorical variables between the groups. In order to investigate the possible attrition bias, all surveys including psychometric questionnaires were compared to respondents who dropped out after baseline or the first follow-up measurement (*n* = 149) on all measurements included in this research.

Descriptive statistics (mean ± standard deviation) of both groups' pre-, post-, and follow-up test results were presented. For these repeated measures, in order to test intra-group differences among these repeated measures, the Friedman test was conducted since normality assumptions were violated. If this test indicated significant differences between the timelines, *post-hoc* analyses were performed using the Wilcoxon signed-rank tests with the Bonferroni correction. Although T1 and T2 were compared over 156 patients in the first research conducted by Bahcivan et al. (2022), in this continuation study the comparisons were made over the remaining 149 patients who participated in the follow-up at 8 weeks. As a result, the differences in gain scores (calculated by subtracting the timelines scores of T2–T1, T3–T1, and T3–T2) between groups were tested using the Mann-Whitney *U* test. The SPSS 25 was utilised for running the analyses; 0.05 was used for the significance “*p”* values. The effect sizes were calculated (Kendall w for Friedman test, ε^2^ for Kruskal–Wallis *H* test, and *r* for Wilcoxon and Mann–Whitney *U* test). The acceptable cut-off for effect size shown by “*w*” and “*r*” values is considered as small (0.10–<0.30), medium (0.30–<0.50), and large (≥0.50) effects. For ε^2^, the values are considered as small (0.01–<0.08), medium (0.08–<0.26), and large (≥0.26) effects.

## Results

Participants' descriptive information can be found in Table 1. Intervention and control groups are of similar characteristics except for the *current city* (*p* > 0.05). In accordance with the main objectives of this study, pre (T1) post (T2) and follow-up (T3) scores were compared and analysed between each other (for intragroup). Later, each of the two measurement timelines was compared separately. The Friedman test found significant differences between these repeated measures in all variables with a generally large effect size for both control and intervention groups shown in Table 2 (*p* < 0.05). In the control group (CG), the *heart rate (HR), anxiety, depression, hopelessness, anger*, and *need help scores* had decreased; however, the *SpO2* and *self-efficacy* scores had increased from T1 to T2. Yet this trend is being maintained from T2 to T3. The *HR, anxiety, depression, hopelessness, anger*, and *need help* scores inclined from T2 to T3 (yet these scores were still lower when comparing with T1), while the *SpO2* and *self-efficacy* scores had declined (yet scores were still higher when comparing with T1).

**Table 1:** Demographic data of the two study groups.

	**Control Group**	**Intervention Group**		
**Variable**	***CBT-CD*** ***n =* 73**	***MBST*** ***n =* 76**	***Total***	***p* value**
Age (years)	52.88 (SD = 9.68)	51.22 (SD = 8.68)	52.03 (SD = 9.19)	0.262^a^
Marital Status				
Single Married	28 (38.4%) 45 (61.6%)	23 (30.3%) 53 (69.7%)	51 98	0.298^b^
Current City				
Izmir Aydin Manisa	28 (38.4%) 31 (42.5%) 14 (19.2%)	47 (61.8%) 12 (15.8%) 17 (22.4%)	75 43 31	<0.001^b^
Treatment Centre				
Hospital	38 (52.1%)	39 (51.2%)	77	0.913^b^
Psychological Consultancy Centre	35 (47.9%)	37 (48.7%)	72	
Living Status Alone W/someone	12 (16.4%) 61 (83.6%)	13 (17.1%) 63 (82.9%)	25 124	0.732^b^
Education Level				
Elementary High School Bachelor or higher	13 (17.8%) 27 (37.0%) 33 (45.2%)	10 (13.2%) 41 (53.9%) 25 (32.9%)	23 68 58	0.115^b^
Employment Status				
Employed Unemployed	37 (50.7%) 36 (49.3%)	28 (36.8%) 48 (63.2%)	65 84	0.089*^b^
Smoking Habit				
Smoker N/Smoker	19 (26.0%) 54 (74.0%)	22 (28.9%) 54 (71.1%)	41 108	0.690^b^
Learning Diagnosis				
1 month < 1–3 Months 3–6 Months 6–Months- 1year 1 year >	2 (2.7%) 13 (17.8%) 15 (20.5%) 20 (27.4%) 23 (31.5%)	4 (5.3%) 8 (10.5%) 14 (18.4%) 13 (17.1%) 37 (48.7%)	6 21 29 33 60	0.139^b^
Metastasis				
Yes No	40 (54.8%) 33 (45.2%)	33 (43.4%) 43 (56.6%)	73 76	0.165^b^

SD, Standard Deviation.

a Mann-Whitney U-test.

b ^χ2^ test.

**Table 2:** Changes in study variables between control and intervention groups among repeated measures (pre, post, follow-up).

**Variables**	**Group**	**Pre test** ** (T1)** ** M (SD)**	**Post test** **(T2)** ** M (SD)**	**Follow-up (T3)** ** M (SD)**	**Friedman test**		**T1–T2**		**T1–T3**		**T2–T3**	
							**Wilcoxon test**		**Wilcoxon test**		**Wilcoxon test**	
					**χ^2^**	**Kendall's W**	**Z**	**r**	**Z**	**r**	**Z**	**r**
HR (bpm)	CG	94.45 (3.31)	92.75 (3.95)	91.40 (5.02)	24.73^*^	0.17	−4.67^**^	0.39	−5.14^**^	0.43	−2.50^**^	0.21
	IG	94.20 (3.86)	85.90 (3.42)	92.24 (4.23)	117.79^*^	0.78	−7.59^**^	0.62	−5.37^**^	0.44	−7.30^**^	0.62
SpO2	CG	94.53 (3.11)	94.66 (3.28)	96.48 (2.77)	47.85^*^	0.33	−0.60	0.05	−5.90^**^	0.49	−5.84^**^	0.48
	IG	95.21 (2.47)	97.13 (1.86)	96.25 (1.93)	52.60^*^	0.35	−6.38^**^	0.52	−3.90^**^	0.32	−3.78^**^	0.52
DISTRESS (ET)	CG	6.29 (1.22)	5.25 (1.01)	4.67 (0.83)	96.67^*^	0.66	−6.86^**^	0.57	−7.07^**^	0.58	−4.19^**^	0.35
	IG	6.26 (1.87)	2.71 (1.71)	5.28 (1.65)	120.38^*^	0.79	−7.38^**^	0.60	−5.08^**^	0.41	−7.15^**^	0.60
ANXIETY												
HADA	CG	12.14 (2.40)	11.37 (1.69)	10.03 (2.63)	32.12^*^	0.22	−3.70^**^	0.31	−5.01^**^	0.41	−3.79^**^	0.31
	IG	12.25 (2.89)	7.08 (3.05)	11.58 (3.42)	114.54^*^	0.75	−7.44^**^	0.60	−2.55^**^	0.21	−7.18^**^	0.60
ANXIETY (ET)	CG	6.29 (1.02)	5.52 (1.18)	4.77 (0.89)	83.49^*^	0.57	−5.20^**^	0.43	−6.94^**^	0.57	−5.31^**^	0.44
	IG	6.54 (1.60)	3.01 (1.72)	5.53 (1.60)	123.78^*^	0.81	−7.37^**^	0.60	−5.41^**^	0.44	−7.09^**^	0.60
STAI	CG	49.02 (5.23)	45.17 (5.93)	43.94 (5.25)	45.38^*^	0.31	−5.72^**^	0.47	−5.77^**^	0.48	−1.76	0.15
	IG	44.99 (5.21)	25.79 (5.95)	44.45 (8.94)	104.05^*^	0.69	−7.50^**^	0.61	−0.15	0.01	−7.46^**^	0.61
DEPRES.												
HADD	CG	12.03 (1.80)	11.55 (2.19)	10.10 (2.56)	23.30^*^	0.18	−2.12^**^	0.18	−4.57^**^	0.38	−3.35^**^	0.28
	IG	11.28 (2.17)	6.50 (2.49)	11.15 (2.67)	113.90^*^	0.75	−7.44^**^	0.60	−0.71	0.06	−7.32^**^	0.60
DEPRES. (ET)	CG	5.73 (0.98)	5.66 (1.06)	4.97 (0.85)	46.74^*^	0.32	−0.57	0.05	−5.14^**^	0.43	−4.98^**^	0.41
	IG	5.79 (1.59)	3.46 (1.47)	5.43 (1.36)	105.02^*^	0.69	−7.01^**^	0.57	−3.13^**^	0.25	−6.87^**^	0.57
SELF-EFFICACY	CG	5.64 (0.77)	5.95 (0.79)	6.57 (0.93)	71.48^*^	0.49	−5.71^**^	0.47	−5.96^**^	0.49	−4.95^**^	0.41
	IG	5.96 (0.90)	8.11 (1.30)	6.49 (1.12)	121.44^*^	0.80	−7.58^**^	0.61	−5.05^**^	0.41	−7.21^**^	0.61
HOPELESNESS	CG	10.11 (2.32)	8.80 (2.31)	5.90 (2.16)	103.02^*^	0.71	−5.75^**^	0.48	−7.15^**^	0.59	−6.03^**^	0.50
	IG	10.58 (3.64)	6.07 (4.21)	7.96 (4.47)	82.17^*^	0.54	−7.44^**^	0.60	−5.69^**^	0.46	−3.98^**^	0.60
ANGER (ET)	CG	5.80 (1.18)	5.45 (1.39)	4.84 (0.96)	33.78^*^	0.23	−2.81^**^	0.23	−5.28^**^	0.44	−3.25^**^	0.27
	IG	5.16 (2.87)	3.09 (2.01)	5.01 (2.33)	72.23^*^	0.48	−6.36^**^	0.52	−0.96	0.08	−6.22^**^	0.52
HELP (ET)	CG	6.85 (1.35)	5.27 (1.60)	5.15 (0.94)	70.61^*^	0.48	−5.43^**^	0.45	−6.86^**^	0.57	−1.17	0.10
	IG	5.82 (2.66)	3.62 (2.15)	5.11 (2.10)	100.86^*^	0.66	−7.07^**^	0.57	−4.53^**^	0.37	−6.47^**^	0.57

* p < 0.05.

** Bonferroni corrected p-value set at p < 0.017.

HR (bpm), Heart Rate (Beats Per Minute); SpO2, Oxygen Saturation Level; HADA, Hospital Anxiety and Depression Scale (Anxiety); HADD, Hospital Anxiety and Depression Scale (Depression); DEPRESS, Depression; STAI, State-Trait Anxiety Inventory; ET, Emotion Thermometer; Pre, Pre-measurement; Post, Post-measurement; Follow-Up, Follow-up measurement.

In order to test whether there were any significant differences, *post-hoc* comparison test was applied by using Wilcoxon test (see Table 2). Additionally, the descriptive statistics of the gain score and the Mann–Whitney *U* test results, which include the comparison of the groups based on the gain score, are presented in Table 3. T1 and T2 results were previously explained by Bahcivan et al. (2022). The comparison between T1 and T3 showed that the method (MBST) used in intervention group (IG) has a significant effect on *HR, SpO*_2_, *Hospital Anxiety and Depression Scale – Anxiety (HADA), ET (distress, anxiety, depression*, and *need for help)* except for *STAI, Hospital Anxiety and Depression Scale – Anxiety (HADD), ET(Anger)*; for the method (CBT-CD intervention) used in control group (CG) has a significant effect in all variables without any exception. Apart from the *HR* scores between ΔT3 and T1, the overall progression was superior in the IG than in CG with small (*SpO2, distress, HADA, depression (ET), Self-efficacy, hopelessness; p* < 0.05, 0.10 < *r* ≤ 0.30) and medium (*STAI, HADD, anger (ET)*, and *need help*; *p* < 0.05, 0.30 < *r* ≤ 0.50) effect.

**Table 3:** Descriptive gain scores and comparison gains scores between control and intervention groups using Mann–Whitney *U*-test.

**Variables**	**Pre/post-test comp**.				**Pre/follow-up test comp**.				**Post/follow-up test comp**.			
	**CBT**	**MSBT**	**Mann–Whitney U**		**CBT**	**MSBT**	**Mann–Whitney U**		**CBT**	**MSBT**	**Mann–Whitney U**	
	**Δ(T2–T1) M (SD)**	**Δ(T2–T1)** ** M (SD)**	**Z**	**r**	**ΔT3–T1 M (SD)**	**ΔT3–T1** ** M (SD)**	**Z**	**r**	**ΔT3–T2 M (SD)**	**ΔT3–T2** ** M (SD)**	**Z**	**r**
HR (bpm)	−1.70 (2.76)	−8.30 (4.16)	−9.05^*^	0.74	−3.06 (4.29)	−1.96 (2.83)	−0.51	0.04	−1.36 (4.70)	6.34 (4.45)	−8.23^*^	0.67
SpO2	0.12 (1.25)	1.92 (1.73)	−6.05^*^	0.50	1.95 (2.07)	1.04 (2.14)	−2.43^*^	0.20	1.82 (2.15)	−0.88 (1.92)	−6.92^*^	0.57
DISTREES (ET)	−1.62 (1.00)	−0.99 (1.35)	−7.85^*^	0.64	−1.62 (0.99)	−0.99 (1.35)	−3.55^*^	0.29	−1.04 (0.77)	−3.55 (2.09)	−9.43^*^	0.77
ANXIETY												
HADA	−0.77 (1.55)	−5.17 (3.24)	−8.35^*^	0.68	−2.11 (2.97)	−0.67 (2.37)	−3.66^*^	0.30	−1.34 (2.84)	4.50 (3.41)	−8.59^*^	0.70
ANXIETY (ET)	−0.77 (1.09)	−3.53 (2.10)	−8.35^*^	0.68	−1.52 (1.00)	−1.01 (1.32)	−3.32^*^	0.27	−0.75 (0.97)	2.51 (2.02)	−9.65^*^	0.79
STAI	−3.85 (4.61)	−19.20 (6.41)	−9.56^*^	0.78	−5.08 (5.90)	−0.54 (7.72)	−4.09^*^	0.34	−1.23 (6.87)	18.66 (9.25)	−9.42^*^	0.77
DEPRES.												
HADD	−0.48 (1.73)	−4.78 (2.68)	−8.72^*^	0.71	−1.93 (3.07)	−0.13 (2.11)	−4.15^*^	0.34	−1.45 (3.32)	4.65 (2.74)	−8.73^*^	0.72
DEPRES. (ET)	−0.07 (0.96)	−2.33 (1.67)	−8.36^*^	0.69	−0.75 (0.98)	−0.36 (0.95)	−3.26^*^	0.27	−0.69 (0.94)	1.97 (1.51)	−9.26^*^	0.76
SELF-EFFICACY	0.31 (0.37)	2.15 (0.90)	−10.02^*^	0.82	0.93 (0.96)	0.53 (0.82)	−3.45^*^	0.28	0.62 (0.98)	−1.62 (1.17)	−9.15^*^	0.75
HOPELESNESS	−1.32 (1.32)	−4.51 (2.62)	−7.42^*^	0.61	−4.21 (2.80)	−2.62 (3.28)	−3.54^*^	0.29	−2.89 (2.97)	1.90 (3.66)	−7.11^*^	0.58
ANGER (ET)	−0.34 (1.15)	−2.07 (1.94)	−6.16^*^	0.50	−0.96 (1.29)	−0.15 (1.19)	−4.05^*^	0.33	−0.62 (1.46)	1.92 (1.85)	−7.63^*^	0.63
HELP (ET)	−1.58 (2.14)	−2.20 (2.25)	−2.16^*^	0.18	−1.70 (1.32)	−0.71 (1.22)	−5.01^*^	0.41	−0.12 (1.55)	1.49 (1.84)	−6.31^*^	0.52

* p < 0.05.

HR (bpm), Heart Rate (Beats Per Minute); SpO2, Oxygen Saturation Level; HADA, Hospital Anxiety and Depression Scale (Anxiety); HADD, Hospital Anxiety and Depression Scale (Depression); DEPRESS, Depression; STAI, State-Trait Anxiety Inventory; ET, Emotion Thermometer; Pre, Pre-measurement; Post, Post-measurement; Follow-Up, Follow-up measurement.

In comparison between T2 and T3, there were significant improvements in all variables except *STAI* and *ET (need for help)* for CG. However, for the IG, the *HR, anxiety, depression, hopelessness, anger*, and *need help* scores had increased; *SpO2* and *Self-efficacy* scores had decreased which was found to be significant (*p* < 0.05). When ΔT3–T2 scores were compared between CG and IG groups, there were significant differences between all variables, and the improvement of CG compared to IG was statistically significant.

In order to observe ΔT3–T1 scores whether they differed statistically using the demographic data that were compared with the Mann–Whitney *U* and Kruskal–Wallis *H* tests from intra-groups (separately for CG and IG) (see Tables 4, 5). Significant differences were observed in *HR, SpO2, hopelessness*, and *anger* variables for both CG and IG in accordance with the *education level*. For *CG, HADA, HADD*, and *IG, STAI* variables were found to have significant differences. According to participants' *marital status*, there is a significant difference in CG only for *self-efficacy* scores. *Living arrangement* and *treatment centres* have no significant impact on any variables. According to *time for learning their diagnosis*, a significant difference was observed in *SpO2, HADD, hopelessness*, and *need help* variables for both CG and IG. In addition to these, the gain scores of *HR* and *HADA* in CG; the *distress, anxiety (ET)*, and *anger* scores were found to have a significant effect on IG. Among those results which were found to be significant, the *distress* and *hopelessness* results for IG have a large effect size, whereas the other variables have a medium effect size. Finally, while *metastatic status* caused a significant difference only in *hopelessness* gain scores for CG, it caused a differentiation in *SpO2, distress, anxiety (ET), hopelessness*, and *anger* gain scores for IG. Of these scores, *anxiety, hopelessness*, and *anger* for IG had a medium effect size, while the others had a minor effect size.

**Table 4:** The impact of demographic variables “a” on anxiety and depression scores, self-efficacy, and hopefulness (ΔT3-T1 scores).

**Variables**	**Group**	**Education level**			**Marital status**			**Living arrangement**		
		**χ^2^^†^**	**ε^2^**	***Post-hoc***	**Z^‡^**	***r***	***Post-hoc***	**Z^‡^**	***r***	***Post-hoc***
HR (bpm)	CG	23.21^*^	0.32	Ps, Hs < Bd^a^	−0.66	0.08	-	−0.64	0.07	-
	IG	13.40^*^	0.18	Bd < Hs^a^	−0.48	0.05	-	−1.07	0.12	-
SpO2	CG	7.46^*^	0.10	Bd < Ps^a^	−1.67	0.20	-	−0.51	0.06	-
	IG	17.87^*^	0.24	Bd < Ps, Hs^a^	−0.28	0.03	-	−1.43	0.16	-
DISTREES (ET)	CG	2.11	0.03	-	−1.41	0.17	-	−1.14	0.13	-
	IG	15.24^*^	0.20	Bd < Hs^a^	−0.24	0.03	-	−0.13	0.01	-
ANXIETY										
HADA	CG	24.44^*^	0.34	Ps,Hs < Bd^a^	−0.95	0.11	-	−1.23	0.14	-
	IG	1.26	0.02	-	−0.53	0.06	-	−0.99	0.11	-
ANXIETY (ET)	CG	3.06	0.04	-	−0.69	0.08	-	−0.13	0.01	-
	IG	3.35	0.04	-	−0.52	0.06	-	−0.91	0.10	-
STAI	CG	4.54	0.06	-	−1.62	0.19	-	−1.13	0.13	-
	IG	9.59^*^	0.13	Hs < Bd^a^	−0.90	0.10	-	−0.98	0.11	-
DEPRES.										
HADD	CG	11.03^*^	0.15	Bd < Ps^a^	−0.44	0.05	-	−0.59	0.07	-
	IG	2.18	0.03	-	−0.37	0.04	-	−1.05	0.12	-
DEPRES. (ET)	CG	9.88^*^	0.14	Bd, Hs < Ps^a^	−0.73	0.09	-	−0.79	0.09	-
	IG	0.89	0.01	-	−0.62	0.07	-	−0.33	0.04	-
SELF-EFFICACY	CG	2.25	0.03	-	−2.51^*^	0.29	S < M^a^	−1.33	0.16	-
	IG	1.06	0.01	-	−1.36	0.16	-	−0.54	0.06	-
HOPELESNESS	CG	17.60^*^	0.24	Bd < Ps, Hs^a^	−0.13	0.01	-	−0.18	0.02	-
	IG	17.69^*^	0.24	Bd < Hs^a^	−0.03	0.00	-	−0.54	0.06	-
ANGER (ET)	CG	6.02^*^	0.08	-	−1.50	0.18	-	−1.16	0.14	-
	IG	9.46^*^	0.13	Bd < Hs^a^	−1.59	0.18	-	−0.77	0.09	-
HELP (ET)	CG	1.04	0.01	-	−0.55	0.06	-	−1.08	0.13	-
	IG	5.66	0.08	-	−0.96	0.11	-	−0.15	0.02	-

* p < 0.05.

a Bonferroni corrected p value.

† Kruskal–Wallis H test;

‡ Mann–Whitney U-test.

PS, Primary School; HS, High School; BD, Bachelor's degree; S, Single; M, Married; HR (bpm), Heart Rate (Beats Per Minute); SpO2, Oxygen Saturation Level; HADA, Hospital Anxiety and Depression Scale (Anxiety); HADD, Hospital Anxiety and Depression Scale (Depression); DEPRESS, Depression; STAI, State-Trait Anxiety Inventory; ET, Emotion Thermometer.

**Table 5:** The impact of demographic variables “b” on anxiety and depression scores, self-efficacy, and hopefulness (ΔT3–T1 scores).

**Variables**	**Group**	**Treatment centre**			**Time for learning their diagnosis**			**Metastatic status**		
		**Z^‡^**	***r***	***Post-hoc***	**χ^2^^†^**	**ε^2^**	***Post-hoc***	**Z^‡^**	***r***	***Post-hoc***
HR (bpm)	CG	−0.50	0.06	-	17.95^*^	0.25	(1 y>) < 1-3 m, 3-6m, 6m-1y	−1.48	0.17	
	IG	−0.90	0.10	-	9.31	0.12	-	−1.18	0.14	
SpO2	CG	−0.26	0.03	-	10.49^*^	0.15	-	−0.44	0.05	
	IG	−0.36	0.04	-	9.75^*^	0.13	-	−2.01^*^	0.23	N < Y
DISTREES (ET)	CG	−0.68	0.08	-	1.88	0.03	-	−0.03	0.00	
	IG	−0.25	0.03	-	22.67^*^	0.30	(< 1 m), 1–3 m < (1 y>)	−2.54^*^	0.29	Y < N
ANXIETY										
HADA	CG	−0.32	0.04	-	13.34^*^	0.19	3–6m < (1 y>)	−0.59	0.07	
	IG	−1.52	0.17	-	6.02	0.08	-	−1.58	0.18	
ANXIETY (ET)	CG	−0.15	0.02	-	6.23	0.09	-	−0.51	0.06	
	IG	−0.49	0.06	-	18.70^*^	0.25	1–3 m < 3-6m, 6m-1y, (1 y>)	−2.65^*^	0.31	
STAI	CG	−0.10	0.01	-	3.73	0.05	-	−0.42	0.05	
	IG	−0.37	0.04	-	6.31	0.08	-	−0.75	0.09	
DEPRES.										
HADD	CG	−0.95	0.11	-	16.06^*^	0.22	(< 1 m) < 6m-1y, (1 y>)	−0.52	0.06	
	IG	−0.42	0.05	-	9.67^*^	0.13	1–3 m < 6m-1y, (1 y>)	−1.57	0.18	
DEPRES. (ET)	CG	−1.01	0.12	-	0.58	0.01	-	−1.08	0.13	
	IG	−1.60	0.18	-	0.62	0.01	-	−1.39	0.16	
SELF-EFFICACY	CG	−1.22	0.14	-	9.02	0.13	-	−1.61	0.19	
	IG	−1.42	0.16	-	5.88	0.08	-	−0.50	0.06	
HOPELESNESS	CG	−0.40	0.05	-	14.02^*^	0.19	(1 y>) < 1-3 m	−2.23^*^	0.26	N < Y
	IG	−0.32	0.04	-	32.51^*^	0.43	(< 1 m), 1-3 m < (1 y>)	−3.33^*^	0.38	Y < N
ANGER (ET)	CG	−1.40	0.16	-	6.69	0.09	-	−1.28	0.15	
	IG	−0.43	0.05	-	17.92^*^	0.24	3-6m < 6m-1y, (1 y>)	−3.27^*^	0.38	Y < N
HELP (ET)	CG	−1.36	0.16	-	13.14^*^	0.18	1-3 m < 6m-1y	−1.05	0.12	
	IG	−1.27	0.15	-	8.05	0.11	-	−0.34	0.04	

* p < 0.05.

^a^Bonferroni corrected p value.

† Kruskal–Wallis H test;

‡ Mann–Whitney U test.

ε^2^ Effect size for Kruskal–Wallis H test; r Effect size for Mann–Whitney U test.

<1 m: less 1 month; 1–3m: 1–3 month; 3–6m: 3–6 month; 6m−1y: 6 month−1 year; 1y >: more than 1 year.

Y, Yes; N, No; HR (bpm), Heart Rate (Beats Per Minute); SpO2, Oxygen Saturation Level; HADA, Hospital Anxiety and Depression Scale (Anxiety); HADD, Hospital Anxiety and Depression Scale (Depression); DEPRESS, Depression; STAI, State-Trait Anxiety Inventory; ET, Emotion Thermometer.

### Tolerability and acceptability of the intervention

There were no significant differences found between the hospital and the private clinic where both MBST and CBT-CD were applied among breast cancer patients (*p* = 0.913). The breast cancer patients who were 18 years old and over could receive both MBST and CBT-CD interventions (*p* > *0.0*5). The MBST intervention benefited women with breast cancer who actively smoked. Additionally, patients' marital status and living arrangements made no difference in receiving MBST (*p* > 0.05). The patients who learned about their cancer diagnosis within more than 1 year showed greater MBST efficaciousness for distress, anxiety (ET), depression (HADD), and hopefulness than patients who learned about their diagnosis for <1–3 months. Patients who had cancer metastasis had no impact on receiving MBST nor CBT-CD intervention (except for distress, hopelessness, and anger) (*p* > 0.05).

## Discussion

This is the first RCT pilot study that investigated the efficacy of Mindfulness-Based Swinging Technique (MBST) for 8 weeks. The present findings indicate that MBST for 20 min single session may be efficacious even after 8 weeks. The participants who received non-repetitive MBST reported significantly reduced perceived stress, anxiety scores, and increased hopefulness. Comparably, non-repetitive 20 min single session of Cognitive Distortion (CD) practise was found to be efficacious significantly for the above-mentioned variables even after 8 weeks. In fact, the CD also reduced depression and state-trait anxiety as well as anger scores for women with breast cancer. Similar to our research, in Solhaug et al. (2019) study, dispositional mindfulness was not measured; therefore, the follow-up results of their study were affected. Solhaug et al. (2016) stated that one's motivation, intention, and attitude in the process of learning mindfulness technique have an impact on the benefit of this intervention. Meaning that, if it measured only the participants who were prone to mindfulness that would violate the randomisation. Perhaps this is why mindfulness practise seems less effective in state-trait anxiety and depression scores after 8 weeks of follow-up. Additionally, Morton et al. (2020) suggest that the objective of further research and clinical practise must focus on improvements in long-term practises, as well as determining the optimal dosage for significant impact on state and trait of mindfulness. Indeed, previous studies (Carlson et al., 2013; Oberoi et al., 2021) stated that patients' knowledge, experiences, and devotion to intervention, along with health-related results, were correlated.

According to Fox (2017), to plan the most suitable treatment for the patient, which ends in more accomplished results, a fully sufficient assessment of the patient's current concerns is the key. Norcross et al. (2013) predicted there would be a growth in short-term (5–12 sessions) and very short-term (1–3 sessions) therapy, in the course of a decrease in long-term (more than 20 sessions) therapy. Otto et al. (2012) stated that it could be difficult for the therapist to bring out the patient's psychological pattern in a short period of time, as 10- to 20-min-long sessions may not be adequate. However, currently, a number of university programs have begun to teach ultra-brief interventions in order to get their mental health students and interns ready, concerning work in mental health settings (Norcross et al., 2013; Sperry and Binensztok, 2019). Therefore, our study compliments such an initiative particularly considering the special needs for women with breast cancer, such as chemotherapy-induced fatigue and cognitive distortions. Thus, it is possible that shorter treatment can persuade more people into health care. Otto et al. (2012) discussed that shortness of treatment duration could be a valid reason why people are less likely to dropout in CBT, compared to different types of psychotherapy. Nonetheless, in our study, the participants' dropout rate was lower in MBST than in CBT-CD. Yet evidence shows that, during the period of tackling psychological challenges, cognitive therapies have a positive impact on patients with breast cancer (Zhang et al., 2016; Xiao et al., 2017).

There are uncertain results when it comes to the effectiveness of mindfulness-based interventions (MBI) compared to CBT. Van Dam et al. (2018) stated that MBI can be effective, whereas Goldin et al. (2016) argue that CBT is superior in particular cases. Goyal et al. (2014) study that has a similar participant size to our study reported that outcomes of trials in breast cancer patients, including randomised and uncontrolled, have indicated that anxiety, depression, and perceived stress were positively affected due to MBI. Since our study's effect size for MBST had dropped after the follow-up, Fjorback et al. (2013)'s study shows similar results. They observed small effects in earlier clinical and non-clinical mindfulness studies that contained briefer follow-up durations. Therefore, a decrease in intervention effects and generally low effect sizes were anticipated (Solhaug et al., 2019).

On the other hand, Solhaug et al. (2019) reported that 36% of participants attended in several different types of mindfulness training, such as qigong, yoga, tai chi, relaxation, and meditation, during the course of the follow-up period. Nevertheless, the outcomes were not dramatically changed even when these participants were eliminated. However, this could be true for the non-cancer participants, this is due to cancer patients who are in active medical treatment may suffer from fatigue that could adversely affect mindfulness practise (Toivonen et al., 2020). Katz and Toner (2013) argued that gender difference is a possible factor that may impact the larger effect sizes amongst female patients with breast cancer. Research shows that women are more prone to utilise mindfulness-based intervention than men (Xunlin et al., 2020). Therefore, this could be one of the possible reasons that our single session of MBST was found to be efficacious even after 8 weeks. Nonetheless, taking “trait and state” into account, there might be a probability that the correlations between independent and dependent variables may be affected by gender as well (Trotman et al., 2019). Anyhow, Trotman et al. (2019) added and indicated that gender differences were not significant when analysing anxiety. It is clear that MBIs are efficacious, but they are not convenient for every person. Mindfulness practise can be difficult in terms of time, directing attention, and also paying full attention throughout meditation according to a systematic review conducted by Tate et al. (2018). Moreover, they discovered that practises may lead to bodily distress and that being a cancer patient resulted in a lot of stressful thinking instead of forgetting about their illness for a while. Therefore, mindfulness techniques can be improved by practise (Baer, 2011; Van Dam et al., 2018).

When it comes to the treatment of various psychological disorders, such as depression, anxiety, and distress, cognitive disputation can be very effective (Sperry and Binensztok, 2019). The concept of effective disputing is that the client gets support from the therapist in order to explore and examine their thinking process so that they are able to provide an acceptable explanation of their automatic thoughts (Lam and Cheng, 1998). This period of time allows them to gain experience, which gives them to an opportunity to understand the incongruity, as well as the illogic of their automatic thoughts, and then develop healthy alternatives (Beck, 2011; Akkoyunlu and Turkcapar, 2013). That might be the reason of single session of 20-min length CBT-CD intervention has efficacious result even after 8 weeks. This is perhaps during the 8-week time, patients have observed the exposed information which was delivered by the therapist (Carona et al., 2021).

The systematic review, conducted by Arab et al. (2016) which focused on HR and breast cancer, was to provide a brief summary of the side effects caused by quite a few breast cancer–related treatments; for instance, comparison of chemotherapy doses, negative effects of the disease resulted exhaustion, low mood, and its connexion to HR. It appears that there was a positive association between mindfulness meditation and decreased post-intervention heart rate, which shows that the mindfulness was adequate (Lorca et al., 2019). That is similar to our MBST intervention where HR was lower in comparison to the post-treatment follow-up. Lastly, Trotman et al. (2019) suggested that it is worth looking into whether there is an association between actual HR or the way one perceives HR alteration and density of anxiety, besides one's perception of anxiety symptoms during the time of psychological stress.

The present pilot study suggests various theoretical, functional, and clinical donations to the developing field of psycho-oncology practise. For instance, a single non-repetitive 20-min session of MBST showed beneficial results in alleviating symptoms of anxiety and stress even after 8 weeks for women with breast cancer who currently receive cancer treatment. Although it was not our intention to test the efficacy of brief CBT-CD intervention; our study uncovered that alternatively breast cancer patients who are in active cancer treatment who were provided with 20-min CBT-CD intervention had 8-week long efficacy in decreasing anxiety, stress, and depression symptoms in some level. This result has a particular significance since psychotherapy sessions that are provided to oncology patients require regular attendance to see a visible outcome. Moreover, our research supports Socratic questioning that is utilised within brief CBT-CD intervention as an efficacious method to be used in psycho-oncological practise which plays a role in closing the gap in the current literature. Furthermore, both CBT-CD and the MBST interventions not only strengthen psychological but also supported physical wellbeing by improving the SpO_2_ level and regulating the HR (bpm) of women with breast cancer. Considering our findings show some encouraging results for the efficacy of a single session of MBST and CBT-CD interventions for 8-week among women with breast cancer. Further research should focus on evaluating these techniques' longer-term effectiveness.

### Limitations, strengths, and future research implications

This study has few limitations. Primarily, patients' attitude about mindfulness or CBT-CD interventions were not measured and randomised regardless. Second, participants' satisfaction level about the MBST intervention is not tested and solely relied on their psychometric outcomes. Third, a researcher had several roles, such as the implementation of the intervention, administration of the questionnaires, and analysis of the data was done by the same person.

On the other hand, there are some strengths of this trial. Firstly, its low attrition rate, with a follow-up rate of 95.51%. The majority of the dropout rates were mainly deceased patients due to severity of their cancer illness (*n* = 7). Our results showed favourable efficacy for one-on-one MBST among women with breast cancer at 8 weeks; therefore, further research evaluating the MBST's longer-term efficacy should be performed for both in group and one-on-one sessions including male breast cancer population as well as patients who were diagnosed with different types of cancer. In fact, due to the possible reduced mobility among cancer patients as well as during the pandemic, it will be particularly valuable to increase online deliverable psycho-oncological interventions; in order to gain more evidence-based psycho-oncological e-health therapies, MBST should be assessed for its online efficacy in integration with virtual reality (VR) tools in future research.

## Data availability statement

The original contributions presented in the study are included in the article/Supplementary materials, further inquiries can be directed to the corresponding author.

## Ethics statement

The studies involving human participants were reviewed and approved by the Research Ethics Committee of Nigde University (Decision Number: 2018/14–01).

## Author contributions

OB has made a substantial, direct, and intellectual contribution. JG-M and TE have equally supervised the entire work and approved it for publication. All authors contributed to the article and approved the submitted version.

## Funding

The authors declare that this study received funding from Oz Bireysel Danismanlik LTD. STI. to cover the publication cost. The funder was not involved in the study design, collection, analysis, interpretation of data, the writing of this article, and the decision to submit it for publication.

## Conflict of interest

Author TE was employed by FEFOC Foundation. The remaining authors declare that the research was conducted in the absence of any commercial or financial relationships that could be construed as a potential conflict of interest.

## Publisher's note

All claims expressed in this article are solely those of the authors and do not necessarily represent those of their affiliated organizations, or those of the publisher, the editors and the reviewers. Any product that may be evaluated in this article, or claim that may be made by its manufacturer, is not guaranteed or endorsed by the publisher.
