# Supplementary material for: A single-session Mindfulness-Based Swinging Technique vs. cognitive disputation intervention among women with breast cancer: A pilot randomised controlled study examining the efficacy at 8-week follow-up
Source: Front Psychol. 2022 Oct 20;13:1007065. doi: 10.3389/fpsyg.2022.1007065 (PMC9632732; doi:10.3389/fpsyg.2022.1007065)
Supplement: Supplementary file 1 [file Table_1.DOCX]

Sample Size:

The sample size was obtained through using the “PS: Power and Sample size” software program - version: 3.1.2, 2014 (Dupont & Plummer, 1991). These calculations were obtained through using the results of STAI score from a previous study based on Turkish cancer population (Alacacioglu et al., 2008). The main reason of using STAI score was due to the higher number of the sample size and the primary outcome was to be calculated based on STAI in comparison with other scales which will be used in this project. This research study of a continuous response variable from independent control and experimental subjects with 1 control per experimental subject.  In the previous study conducted with breast cancer patients in Turkey; the response within each subject group was normally distributed with standard deviation 8,7 (Alacacioglu et al., 2008). Based on the results of previous studies (Lengacher et al., 2016; Zhang, Zhou, Feng, Fan, Zeng & Wei, 2016; Sharplin, Jones, Hancock, Knott, Bowden, & Whitford, 2010) which were conducted to evaluate the effectiveness of mindfulness intervention on cancer patients’ anxiety whether the true difference in the experimental and control means is 6. The mean score of 6 is found by observing the previous studies’ comparison scores of both experimental and control groups (Lengacher et al., 2016; Zhang et al., 2016, Sharphin et al., 2010).

Then for this study the necessary subjects’ numbers will be 45 for experimental, and 45 for control group to be able to reject the null hypothesis that the population means of the experimental and control groups are equal with probability (power) 0,9. The Type I error probability associated with this test of this null hypothesis is 0,05. However, it will be aimed to recruit 70 participants in each group; in case of there will be losses or disengagements about the participants.
